# Unpaired Underwater Image Synthesis with a Disentangled Representation for Underwater Depth Map Prediction

**DOI:** 10.3390/s21093268

**Published:** 2021-05-09

**Authors:** Qi Zhao, Zhichao Xin, Zhibin Yu, Bing Zheng

**Affiliations:** 1 College of Information Science and Engineering, Ocean University of China, Qingdao 266100, China; zq4984@stu.ouc.edu.cn (Q.Z.); xinzhichao@stu.ouc.edu.cn (Z.X.); bingzh@ouc.edu.cn (B.Z.); 2 Sanya Oceanographic Institution, Ocean University of China, Sanya 572000, China

**Keywords:** underwater image synthesis, underwater depth map estimation, image-to-image translation

## Abstract

As one of the key requirements for underwater exploration, underwater depth map estimation is of great importance in underwater vision research. Although significant progress has been achieved in the fields of image-to-image translation and depth map estimation, a gap between normal depth map estimation and underwater depth map estimation still remains. Additionally, it is a great challenge to build a mapping function that converts a single underwater image into an underwater depth map due to the lack of paired data. Moreover, the ever-changing underwater environment further intensifies the difficulty of finding an optimal mapping solution. To eliminate these bottlenecks, we developed a novel image-to-image framework for underwater image synthesis and depth map estimation in underwater conditions. For the problem of the lack of paired data, by translating hazy in-air images (with a depth map) into underwater images, we initially obtained a paired dataset of underwater images and corresponding depth maps. To enrich our synthesized underwater dataset, we further translated hazy in-air images into a series of continuously changing underwater images with a specified style. For the depth map estimation, we included a coarse-to-fine network to provide a precise depth map estimation result. We evaluated the efficiency of our framework for a real underwater RGB-D dataset. The experimental results show that our method can provide a diversity of underwater images and the best depth map estimation precision.

## 1. Introduction

In 3D computer vision, a depth map refers to a frame in which each pixel represents the distances of the surfaces of objects in a scene from a viewpoint. There are a number of uses for depth maps, including machine vision, 3D reconstruction, and shadow mapping [1]. As an important branch of underwater vision, underwater depth map estimation plays an important role in many fields, including underwater landform surveys, vehicle navigation, and underwater hull cleaning. Although considerable progress has been achieved in screening-laser-technology-based underwater 3D reconstruction [2], many approaches have the limitation that the patterns cannot be changed online [3]. In addition, calibration-based methods can be affected by the index of refraction transformation [4]. Some in-air depth map estimation devices, such as the Kinect [5], Lidar [6], or monocular lenses [7], can only obtain a limited effect in an underwater environment [8]. The major challenge comes from the complicated underwater environment. Most underwater images are captured with low contrast due to the scattering and absorption degradation caused by underwater particulates [9]. Inhomogeneous illumination further intensifies the problem of color distortion in underwater images.

While deep-learning-based methods have achieved great success in the field of computer vision [10,11], the progress is still considerably limited in the field of image-based underwater depth map estimation. The lack of data is a major challenge when deploying a deep learning model with supervised learning for underwater depth map estimation. Collecting underwater images is expensive and time consuming, as is the collection of paired underwater RGB-D data containing underwater images and corresponding depth maps. The success of generative adversarial networks (GANs) in the field of image-to-image translation [12,13,14,15] provides a feasible way to translate images between two domains or multiple domains in an unsupervised manner.

At present, many researchers are attempting to synthesize underwater images with in-air RGB-D images to build paired datasets for underwater image color restoration [16,17,18] or depth map estimation [10,11,19]. For instance, WaterGAN [16] and UWGAN [20] input a paired in-air RGB-D image into a physical-model-based generator such that the final output is a synthesized underwater image produced by the generator [10,11]. However, these methods adopt a two-stage training strategy in which the modules for underwater depth map estimation and synthesis of underwater images are isolated, thus ignoring the latent relationship between visual images and depth information.

In a recent work, a method called UW-Net [11] was constructed in a single-stage network with two generators to simultaneously synthesize an underwater image and estimate an underwater depth map. However, all of these models attempted to build a function for mapping from the synthetic images to the target domain by using one single network, which led to poor performance in terms of both depth map estimation and image synthesis tasks. Moreover, none of the methods mentioned above could generate various underwater images with disentangled representations, which may lead to an inefficient use of training data and a lack of diversity in underwater image synthesis. In order to solve these problems, we propose a novel image-to-image translation framework for underwater image synthesis and depth map estimation. A discussion of our motivations is presented in the following.

In practice, it is relatively easy to obtain unlabeled underwater images from the internet. These images may include rich information on various underwater conditions, which may help our synthetic framework in generating underwater images with a rich diversity. However, labeling these images is a time-consuming task. Inspired by the success of InfoGAN [21] and its extensions [22], we redesigned the loss functions of our framework to include interpretative disentangled representations of various underwater conditions, including the illumination and water color.

**Figure 1:**
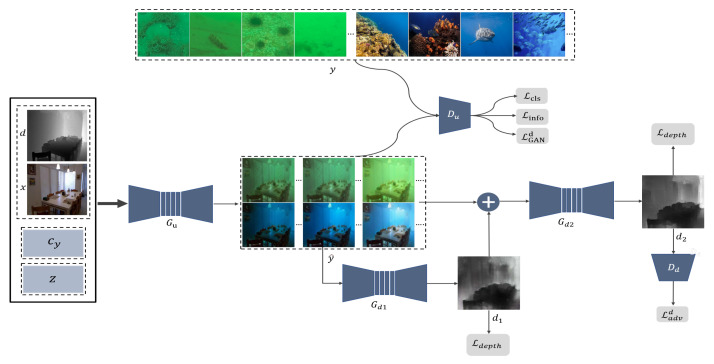
The network framework of our proposed model was designed to synthesize multiple underwater images and estimate underwater depth maps. We used the generator $G_{u}$ and the discriminator $D_{u}$ to synthesize various underwater images in the given underwater domain $c_{y}$. We designed the generators $G_{d1}$ and $G_{d2}$ and the discriminator $D_{d}$ to learn to estimate underwater depth maps based on the synthesized underwater RGB-D dataset.

Due to the decreased visibility and lack of references, another practical problem of our underwater depth map estimation task is that objects at different distances cannot show uniformly show precise information. Therefore, we adopted a multi-depth estimator mechanism to accomplish coarse-to-fine adjustment. As Figure 1 shows, our two depth generators are responsible for the global–coarse depth map estimation and local–fine depth map estimation, respectively. With the depth map passing though these two generators, depth information is refined and forces the generators to pay attention to nearby objects. Overall, the main contributions of this paper are summarized as follows:

- We propose a novel end-to-end framework that applies image-to-image translation to underwater depth map estimation and further boosts current underwater depth map estimation research.
- To enrich our synthesized underwater dataset, we propose a disentangled representation loss along with style diversification loss to identify interpretable and meaningful representations from the unlabeled underwater dataset and the synthesized underwater images with a rich diversity.
- Following the coarse-to-fine principle, and inspired by the work of Eigen et al. [23] and Skinner et al. [19], our approach adopted global–local generators for the estimation of coarse and fine depth maps, respectively. We evaluated our model on a real underwater RGB-D dataset and achieved better results than those of other state-of-the-art models.

## 2. Methods

### 2.1. Overall Framework

Because supervised learning could not be directly performed due to the lack of paired underwater RGB-D images, we designed a two-stage model, as described in Figure 1. Our model includes two cascades: an underwater image synthesis module and an underwater depth map estimation module. The first underwater image synthesis module can translate an original in-air image with its corresponding depth into the underwater domain with disentangled representations to generate various underwater RGB-D pseudo-pairs. The synthetic pseudo-pairs were further used to provide the underwater depth map estimation module with supervised learning through a coarse-to-fine process. Our overall framework consists of three generators, namely, $G_{u}:\left(x,d,c_{y},z\right)\to \tilde{y}$, $G_{d1}:\tilde{y}\to d_{1}$, and $G_{d2}:\left(\tilde{y},d_{1}\right)\to d_{2}$, where x represents the original in-air images, d is the corresponding depth map, $c_{y}$ is the target underwater domain, z is the continuous noise vector, $\tilde{y}$ is the generated underwater image, $d_{1}$ represents the global results of the underwater depth map estimation, and $d_{2}$ is the final estimated depth map. According to the two tasks, we also designed two discriminators, $D_{u}$ and $D_{d}$. $D_{u}$ aims to distinguish real and fake underwater images and classify their corresponding domains in the real and fake underwater images. The discriminator $D_{d}$ only aims to distinguish real and fake underwater depth maps.

**Underwater image synthesis with disentangled representation**. We referred to StarGAN [15] and InfoGAN [21] to design the underwater image synthesis module. We defined a random noise vector (*z*) and target domain label vector ($c_{y}$) to produce multiple outputs in a specific domain. To ensure that the generated underwater images preserved the original depth information after translation, the inputs of our module included four parts, namely, the in-air image (x), the corresponding depth (d), the target underwater label ($c_{y}$), and the noise vector (z), to synthesize an underwater image $\tilde{y}$ = $G_{u}\left(C\left(x,d,c_{y},z\right)\right)$, where *C* represents depth-wise concatenation. The generator $G_{u}$ was taken from CycleGAN [12] and StarGAN [15]. To guarantee that the synthetic image $\tilde{y}$ belonged to the target domain $c_{y}$, we designed the discriminator $D_{u}$ by following the PatchGAN [13] with three branches (domain classification, computation of naturalness, and limit of the coupling of noise (Z)). The domain classification loss $L_{cls}$ was designed for the classification task of recognizing the underwater domain attributions ($c_{y}$) of the synthesized image $\tilde{y}$ and real underwater images *y*. Notably, *y* did not have the corresponding depth annotation due to the lack of an underwater ground truth. Furthermore, to force the noise vector *z* to represent and control the disentangled information from the underwater environment, we also defined an auxiliary discriminator *Q*, which refers to InfoGAN [21].

**The coarse-to-fine underwater depth map estimation process**. According to the characteristics of underwater depth map estimations, we designed a coarse-to-fine generative adversarial network that includes two identical generators, $G_{d1}$ and $G_{d2}$. Following the work on UW-Net [11], we also chose DenseNet [24] for the generators. Differently from UW-Net [11], each dense block [24] has five layers with eight filters. In the training stage, we took the synthetic underwater images $\tilde{y}$ from the synthetic module as the input of the coarse network $G_{d1}$. To obtain a broadly correct result, we adopted the $L_{1}$ norm, which makes equal contributions to distant and nearby points in a scene. Then, the output of the coarse generator $G_{d1}$($\tilde{y}$) and the generated underwater images $\tilde{y}$ were used as the input of the fine generator $G_{d2}$ to obtain a better depth map $G_{d2}\left(C\left(G_{d1}\left(\tilde{y}\right),\tilde{y}\right)\right)$. Unlike the coarse prediction task in $G_{d1}$, we also introduced the $L_{depth}$ loss to guide the fine generator $G_{d2}$ for more in-depth observations. Specifically, the discriminator $D_{d}$ was a PatchGAN [13] with only one discrimination output.

### 2.2. Loss Functions

**Adversarial Loss**. As an extension of a conditional GAN, the conditional generative adversarial loss [25] was used as a basic component of our loss functions. During the training process, the generator $G_{u}$ took hazy in-air RGB-D image pairs (x, d), the target domain label $c_{y}$, and the continuous noise vector *z* as inputs, and it learned to generate underwater images $G_{u}$(x, d, $c_{y}$, z) through adversarial loss [26]. $L_{GAN}^{u}$ can be expressed as follows:

(1) $$\begin{array}{cc} L_{GAN}^{u} & =\underset{G}{min}\;\underset{D}{max}\{E_{x,y∼P_{data}\left(x,y\right)}\left[{\left(D_{u}\left(y\right)-1\right)}^{2}\right]\\ \; & +E_{x∼P_{data}\left(x\right)}\left[\left(D_{u}{\left(\tilde{y})\right)}^{2}\right]\right\},\\ \; & where\;\tilde{y}=G_{u}\left(C\left(x,d,c_{y},z\right)\right), \end{array}$$

where $G_{u}$ aims to synthesize the multiple underwater images $G_{u}\left(C\left(x,d,c_{y},z\right)\right)$ belonging to the target domain $c_{y}$. The discriminator $D_{u}$ learns to distinguish the real underwater image *y* and the synthesized underwater image $\tilde{y}$. For underwater depth map estimation, the adversarial loss $L_{GAN}^{d}$ is described as:

(2) $$\begin{array}{cc} L_{GAN}^{d} & =\underset{G}{min}\;\underset{D}{max}\{E_{\tilde{y},d∼P_{data}\left(\tilde{y},d\right)}\left[{\left(D_{d}\left(d\right)-1\right)}^{2}\right]\\ \; & +E_{\tilde{y}∼P_{data}\left(\tilde{y}\right)}\left[{\left(D_{d}\left(d_{2}\right)\right)}^{2}\right]\},\\ \; & where\;d_{2}=G_{d2}\left(C\left(G_{d1}\left(\tilde{y}\right),\tilde{y}\right)\right), \end{array}$$

where the $G_{d1}$ output is a global depth map $d_{1}$ from the synthesized underwater images $\tilde{y}$. Based on the output of $G_{d1}$, $G_{d2}$ attempts to fine-tune the results. $D_{d}$ learns to recognize the estimated depth output $d_{2}$ from the inputs.

Feature-matchingloss. In the process of underwater image synthesis, to preserve the object content of the original in-air images and to pair the contents of the synthesized underwater images and their corresponding in-air depth maps, a feature-level loss function [14,27] was introduced, which is called $L_{feat}$. The loss is based on a pre-trained VGG19 network [28] that extracts the feature representations from fake and real underwater images. It can effectively preserve the content of the objects between the original images *x* and the generated underwater images $\tilde{y}$. Moreover, it only changes the domain-related parts of the original images and does not have any negative effects on underwater image synthesis. $L_{feat}$ is expressed as follows:

(3) $$L_{feat}=\sum_{i=0}^{N}\frac{1}{M_{i}}\left[|\right|\Phi^{\left(i\right)}\left(x\right)-\Phi^{\left(i\right)}\left(G_{u}\left(x,d,c_{y},z\right)\right){\left|\right|}_{1}].$$

where $\Phi^{\left(i\right)}$ denotes the feature maps at the *i*-th layer with $M_{i}$ elements of a pre-trained VGG19 network [28]. The parameters that we set can be found in the work of Kupyn et al. [29].

**Domain classification loss**. Our model aims to generate multi-style underwater images and continuous outputs in a given underwater style. It involves two domain classification losses: discrete domain classification loss and continuous domain classification loss. Here, the domain classification loss is used to classify discrete domains. Inspired by UMGAN [10] and StarGAN [15], we included an optional domain classification loss to handle a classic domain classification task, which forces the synthetic sample $\tilde{y}$ to be generated in the target domain $c_{y}$. The domain classification loss $L_{cls}^{r}$ is defined as follows:

(4) $$\begin{array}{c} L_{cls}^{r}=E_{y,c^{\prime }}\left[-\log D_{u}\left(c^{\prime }|y\right)\right]. \end{array}$$

where the discriminator $D_{u}$ learns to classify the real underwater images to their original domain *c*′. For generator $G_{u}$, the loss function for the domain classification of the synthetic underwater images is defined as:

(5) $$\begin{array}{c} L_{cls}^{f}=E_{\tilde{y},c_{y}}\left[-\log D_{u}\left(c_{y}|\tilde{y}\right)\right]. \end{array}$$

where the discriminator $D_{u}$ attempts to classify the generated underwater images to their target underwater domain $c_{y}$.

**Disentangled representation loss**. To output continuous underwater images in a given underwater style, a continuous domain classification loss—namely disentangled representation loss—was designed. Inspired by InfoGAN [21], we included the disentangled representation loss to make the generator $G_{u}$ extract various representations from real underwater images with a random noise vector *z*. The vector *z* could be set to either a binary or a decimal value according to the different tasks. In the test stage, the generator $G_{u}$ could generate a controllable synthetic underwater image $\tilde{y}$ by using a specified latent vector *z*. The disentangled representation loss $L_{info}$ can be expressed as:

(6) $$\begin{array}{c} L_{info}=\left[|\right|Q_{u}\left(\tilde{y}\right)-{z||}_{2}]. \end{array}$$

Similarly to the model setting in InfoGAN, here, $Q_{u}$ is a sub-network of the discriminator $D_{u}$.

**Style diversification loss**. As a supplement to the disentangled representation loss, we referred to StarGANv2 [30] and the style diversification loss $L_{dis}$ to maximize the intra-domain distance in order to stabilize the training process and produce various outputs for a given input image pair (x, d) in a target domain $c_{y}$. We maximized the loss term and minimized the info loss force of $G_{u}$ to generate multiple controllable underwater images in a given domain. The style diversification loss $L_{dis}$ can be written as follows:

(7) $$\begin{array}{c} L_{dis}=\left[|\right|G_{u}\left(x,d,c_{y},z_{i}\right)-G_{u}\left(x,d,c_{y},z_{j}\right){\left|\right|}_{1}]. \end{array}$$

where $z_{i}$ and $z_{j}$ represent the latent vectors of two samples.

**Reconstruction loss**. For unpaired image-to-image translation, the cycle consistency loss [12] is commonly used to preserve domain-invariant characteristics and stabilize the training process. In our model of underwater image synthesis, the reconstruction loss $L_{rec}$ between the hazy in-air images x and reconstructed image $\tilde{x}$ is defined as follows:

(8) $$\begin{array}{c} L_{rec}=E_{x,c_{y},c_{x}}[||x-\hat{x}{\left|\right|}_{1}],\\ \hat{x}=G_{u}\left(C\left(G_{u}\left(C\left(x,d,c_{y},z\right)\right),d,c^{\prime },z\right)\right), \end{array}$$

Depthloss. Our coarse network $G_{d1}$ estimates a global and coarse depth map $d_{1}$ from the generated underwater image $\tilde{y}$. Here, we adopted the general $L_{1}$ norm between the generated depth map $d_{1}$ and its ground truth d. The $L_{1}$ norm has an equal contribution between distant and nearby points in a scene. Separately, the fine network should pay more attention to nearby points [31]. Therefore, we explored a loss to guide our coarse-to-fine network. So, the loss $L_{depth}$ can be expressed as follows:

(9) $$\begin{array}{c} L_{depth}=\left[|\right|G_{d1}\left(\tilde{y}\right)-{d||}_{1}+\frac{1}{n}\sum_{n}^{i=1}ln(||d_{2}-{d||}_{1}+1)],\\ d_{2}=G_{d2}\left(C\left(G_{d1}\left(\tilde{y}\right),\tilde{y}\right)\right), \end{array}$$

where $G_{d1}$ tries to globally estimate the depth map from the generated underwater images $\tilde{y}$. $G_{d2}$ tries to locally fine-tune the depth map $d_{1}$. The final results are $d_{2}$ after fine-tuning.

**Full objective**. Our full objective functions can be written as follows:

(10) $$L_{D_{u}}=L_{GAN}^{u}+\alpha L_{cls}^{r}$$

(11) $$L_{G_{u}}=L_{GAN}^{u}+\alpha L_{cls}^{f}+\eta L_{feat}+\gamma L_{info}-\theta L_{dis}+\beta L_{rec}$$

(12) $$L_{D_{d}}=L_{GAN}^{d}$$

(13) $$L_{G_{d}}=L_{GAN}^{d}+\lambda L_{depth}$$

where α, η, γ, θ, β, and λ are the hyperparameters for each term. We optimized these parameters with a greedy search and set α=1,η=1,γ=0.1,θ=0.1,β=1, and λ=50 in all of our experiments. The optimization of our model was successful.

## 3. Results

### 3.1. Datasets and Implementation Details

Our experiments mainly involved two tasks: underwater image synthesis and underwater depth map estimation. For the first task, we synthesized underwater images from hazy in-air RGB-D images and evaluated the image qualities with multiple image generation models, including WaterGAN [16], CycleGAN [12], StarGAN [15], UW-Net [11], and NICE-GAN [32]. For the second task, we evaluated our depth map estimation results with a real underwater RGB-D dataset. We compared the depth map estimation results obtained using the methods of dark channel prior (DCP) [33], underwater dark channel prior (UDCP) [34], Berman et al. [35], and Gupta et al. [11], as well as our method of underwater depth map estimation. Following the experimental setting of UW-Net [11], we also chose the D-Hazy dataset [36] as the in-air RGB-D images for the inputs. Note that both UW-Net and our model can be fine-tuned on the dataset of Berman et al.. The real underwater datasets for training contained 1031 blue and 1004 green underwater images from the SUN [37], URPC (http://www.cnurpc.org/ (accessed on 5 August 2019)), and Fish datasets (http://www.fishdb.co.uk/ (accessed on 7 October 2018)). We randomly chose 1400 images for the training dataset from the D-Hazy dataset [36], which includes 1449 paired in-air RGB-D images. The remaining pairs were used for evaluation. We took 128×128 patches for training and 256×256 complete images for testing. The training took about 40 h on one Nvidia GeForce GTX 1070 (8GB) using the Pytorch framework. To avoid mode collapse, we also introduced spectral normalization [38]. Following the work of BigGAN [39] and SAGAN [40], the learning rates were set to 0.0002 in the discriminators and 0.00005 in the generators. We set the batch size to 10, and the model was trained for 80,000 iterations in our experiments.

#### 3.1.1. Qualitative Evaluation

**Figure 2:**
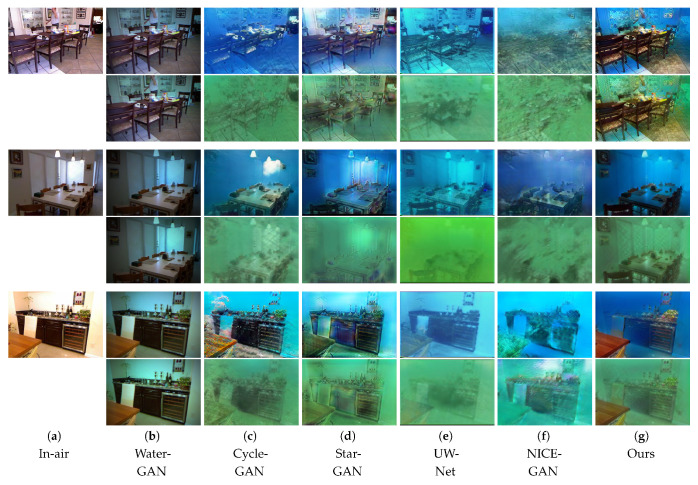
Comparison of the visual quality of the synthetic underwater images using the following methods: WaterGAN [16], CycleGAN [12], StarGAN [15], UW-Net [11], NICE-GAN [32], and our method.

**Figure 3:**
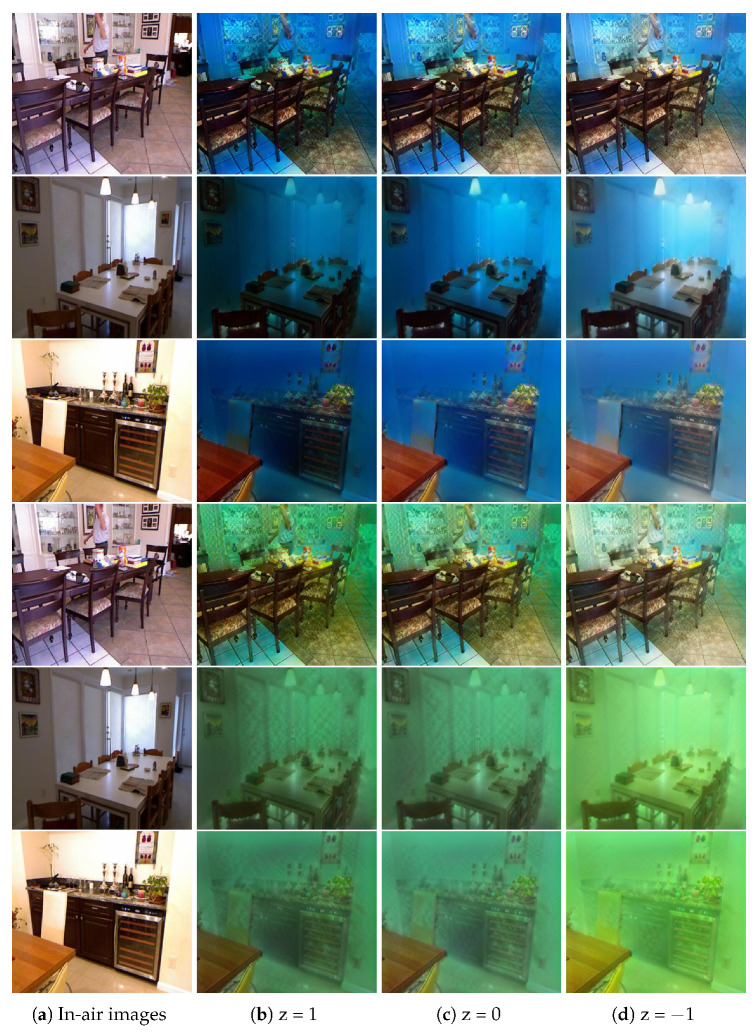
Sample results of our method for underwater image synthesis. The continuous noise *z* was used to generate multiple underwater images with a specific domain. (**a**) In-air images, (**b**–**d**) multiple underwater images generated in two specific domains (blue and green).

To evaluate the effectiveness of the synthetic underwater images, we compared our method with other approaches on the NYU v2 [41] and D-Hazy datasets [36]. To show how close our synthetic images were to the real underwater images, we present some synthetic images in Figure 2. WaterGAN [16] refers to the underwater imaging process and takes in-air RGB-D images as input to synthesize underwater images. As shown in Figure 2b, the results of WaterGAN [16] are close to the in-air images and lack underwater characteristics. In Figure 2c, the underwater images generated by CycleGAN [12] seem better than those of WaterGAN [16]. However, the results of CycleGAN [12] include serious structural distortions, such as the vase in the fifth row of Figure 2c. StarGAN [15] can simultaneously synthesize multi-style underwater images (Figure 2d), but the results still do not meet expectations due to the lack of depth information and clear structural information. In addition, the results retain many artifacts, such as the desk in the last row of Figure 2d. To retain the depth information for better underwater depth map estimation, UW-Net [11] takes the hazy in-air RGB-D images as input and uses DenseNet [24] for the generators, as shown in Figure 2e; this method shows a fuzzy structure. The results of NICE-GAN [32] can be seen in Figure 2f, and there are many artifacts in the results. Furthermore, most of the methods, including WaterGAN [16], CycleGAN [12], UW-Net [11], and NICE-GAN [32], are in two domains, and only StarGAN [15] can synthesize multi-style images. None of the above-mentioned methods consider the diversities in a given style. The synthetic underwater images from our method are shown in Figure 2g; the structure and depth information is well preserved. Our methods can simultaneously synthesize multi-style underwater images and use the noise *z* to produce multiple outputs with a target style, as shown in Figure 3. Here, we set z = 1, 0, −1. Overall, for underwater image synthesis, our method performed better and generated more diverse outputs than the other methods.

**Figure 4:**
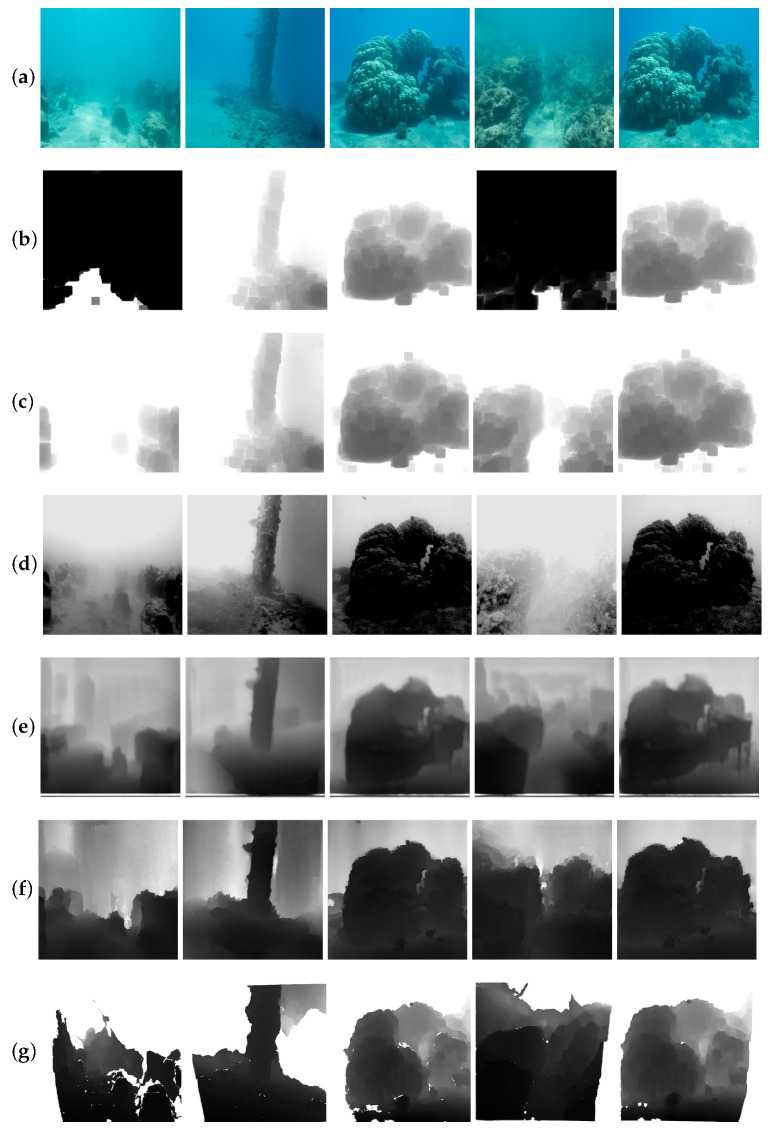
Comparison of our method with other methods for underwater depth map estimation. (**a**) Teal underwater images. (**b**–**g**) Results of DCP [33], UDCP [34], Berman et al. [35], UW-Net [11], and our method, as well as the ground truth.

**Figure 5:**
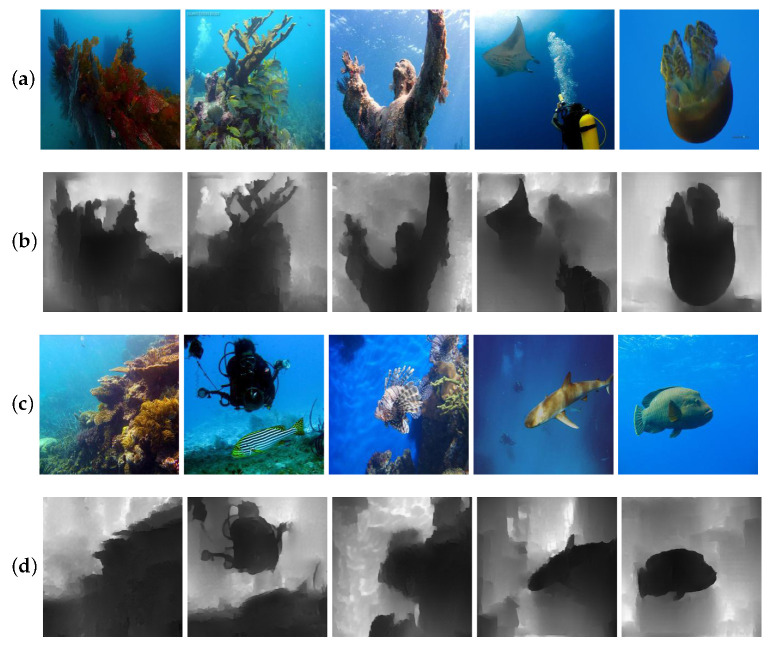
Estimation of multiple underwater depth maps. (**a**,**c**) are real underwater images. (**b**,**d**) are their predicted depth maps. Note that there is no ground truth.

Following the work of UW-Net [11], we used the dataset from Berman et al. [35] to compare our method with other methods. Some results are shown in Figure 4. The former three methods are based on traditional physical processes that rely on pre-estimated parameters. Comparing them with the deep-learning-based UW-Net [11] and our method, we note that the latter two were able to obtain depth maps with smoother predictions. The predicted depth map of our method seems to be more accurate than that of UW-Net [11]. More qualitative results can be seen in Figure 5.

#### 3.1.2. Quantitative Evaluation

To quantitatively evaluate our model, we adopted two metrics for comparison: log scale-invariant mean squared error (SI-MSE [1]) and the Pearson correlation coefficient (ρ) with the dataset from Berman et al. [35]. Higher ρ values and lower SI-MSE [1] values represent better results. Due to the limitations of the Berman dataset, the ground truth was not fully provided in each depth map. We only evaluated the pixels with a distance value that was defined in the ground truth (GT). Comparing our method with other approaches, namely, DCP [33], UDCP [34], Berman et al. [35], and UW-Net [11], we observed that our method obtained the lowest scale-invariant error (SI-MSE [1]) and the highest Pearson correlation coefficient (ρ) (Table 1).

### 3.2. Ablation Study

The lack of diversity is the main obstacle in obtaining a precise underwater depth map with a data-driven model. We believe that the disentangled representation and the coarse-to-fine strategy play key roles in increasing the diversity of synthetic underwater images and enhancing the depth map prediction results. We evaluated the effectiveness of each proposed component, as shown in Table 2. Our framework included the underwater image synthesis module and the underwater depth map estimation module. Theoretically, underwater image synthesis with disentangled representation can be used to generate realistic underwater images that are rich in diversity. A coarse-to-fine pipeline can further help our model to obtain better estimation results. From Table 2, we can observe that synthesizing multiple underwater images with disentangled representation and adopting a coarse-to-fine pipeline can practically help our model to obtain the best scores for SI-MSE and ρ in the final depth map estimation task.

## 4. Discussion

### 4.1. Cross-Domain Underwater Image Synthesis with Disentangled Representation

**Figure 6:**
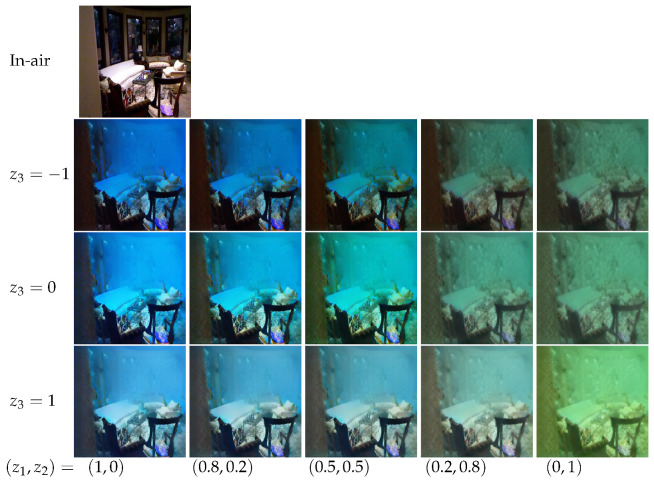
The continuous process of synthesizing underwater images. We used pseudo-labels to represent domain attributes. The two dimensions represent the green style (deep green when $z_{1}=0,z_{2}=1$) and blue style (deep blue when $z_{1}=1,z_{2}=0$), respectively. The first row on the top is the source in-air image, and the remaining images show the gradual transition from the blue style to the green style with different latent variables $z_{3}$.

In this section, we further explore the potential of our model for underwater image generation. With the help of the disentangled representation loss, our model can generate the intermediate information between two domains with semi-supervised learning. In this experiment, we removed the discrete conditional vector $c_{y}$. Instead, we assigned a three-dimensional vector $\left(z_{1},z_{2},z_{3}\right)$ with decimal values for our task, where $z_{1}$ and $z_{2}$ were used for semi-supervised learning to control the underwater color, and $z_{3}$ was a free latent variable. To control the synthesized water color in a continuous manner, we manually labeled 20% of the underwater images from each underwater domain (blue and green). The deep blue images are labeled (1, 0), and the deep green images are labeled (0, 1). Both the labeled (20%) and unlabeled (80%) underwater images were used for training. The unlabeled underwater images were labeled by the classification branch from the discriminator $D_{u}$, which was introduced in Section 2.1. The results are shown in Figure 6. We can observe that our model can perform a gradual transition from the blue style to the green style according to the values of $z_{1}$ and $z_{2}$. Without any ground truth for the illumination, we found that our model could also perform a gradual transition from dark to bright according to the value of the free latent variable $z_{3}$.

**Figure 7:**
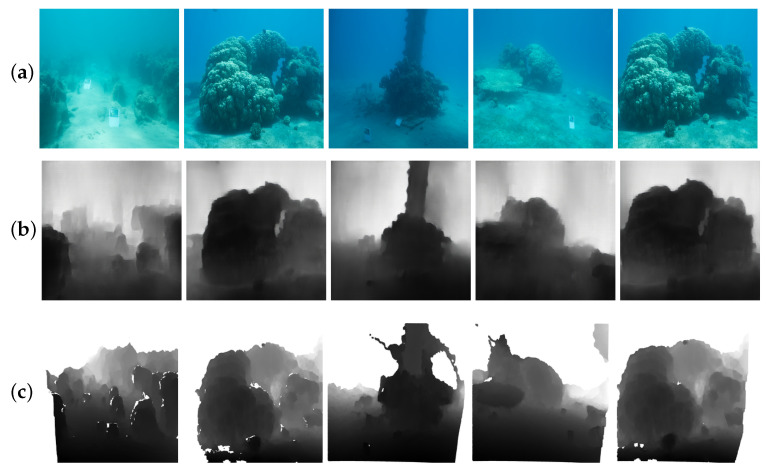
Sample results of our method using pseudo-labels for underwater depth map estimation. (**a**) Real underwater images from the dataset provided by Berman et al. [35]. (**b**,**c**) are the results of our model for depth map estimation and the ground truth.

We also evaluated the effectiveness of the synthesized underwater images for underwater depth map estimation, as shown in Figure 7. The quantitative results can be found in Table 3. The experiments show that the cross-domain synthetic strategy can also practically improve the performance in underwater depth map estimation. Our model with the cross-domain synthesis (Ours-C) setting obtained a lower SI-MSE score and an improved ρ score compared to Berman et al.’s dataset, which indicates that the cross-domain synthesis task can practically increase the diversity of the synthetic images and the generalization ability of our model. Although both models (Ours-C(fine-tuned (FT)), Ours(FT)) had a similar performance when they were fine-tuned on the unlabeled test dataset, note that one might not always have the opportunity to obtain a test dataset before deploying the model.

### 4.2. Challenges of Underwater Scenes with Inhomogeneous Illumination

**Figure 8:**
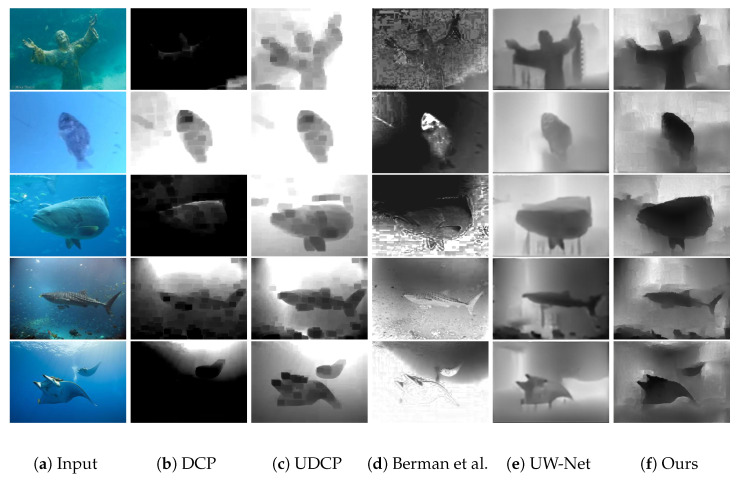
Comparison of the results of underwater depth map estimation in various underwater images with bad visibility by using different methods. We compared the results of dark channel prior (DCP) [33], underwater dark channel prior (UDCP) [34], Berman et al. [35], and Gupta et al. [11] with those of our method.

Due to the reflections and the angular changing of illuminants, many real underwater images show bad visibility with inhomogeneous illumination, as seen in Figure 8a. These factors usually bring negative effects for detection, segmentation, and depth map estimation in real underwater images. The inhomogeneous illumination can easily cause a domain shift and mislead the feature extraction process. As seen in Figure 8, we show some results of DCP [33], UDCP [34], Berman et al. [35], UW-Net [11], and our method. As seen in the first two rows of Figure 8, the objects are difficult to accurately recognize from the real underwater images, which have a low contrast. Compared to the other methods, our model has a lower error ratio. However, our model still achieves inaccurate background depth map prediction results. Domain adaptation [42] might be a solution for improving our model in order to overcome this obstacle. We will consider this in our future research.

## 5. Conclusions

In this paper, we proposed an end-to-end system for underwater image synthesis and underwater depth map estimation. Our model can synthesize underwater images in a continuous manner to construct RGB-D pairs with disentangled representations. The coarse-to-fine pipeline can practically increase the performance for the task of underwater depth map estimation. We adopted a series of experiments for comparisons with the existing state-of-the-art methods. Both qualitative and quantitative results proved the efficiency of our method in both tasks.

## Figures and Tables

**Table 1:** Quantitative comparison of our method and other methods on the dataset of Berman et al. [35]. FT represents a fine-tuned (FT) underwater model. Lower SI-MSE [1] values and higher ρ values are better for underwater depth map estimation.

	DCP	UDCP	Berman et al.	UW-Net	UW-Net (FT)	Ours	Ours (FT)
SI-MSE	1.3618	0.6966	0.6755	0.4404	0.3708	0.3199	**0.2486**
ρ	0.2968	0.4894	0.6448	0.6202	0.6451	0.7018	**0.7600**

**Table 2:** Ablation study of our method.

	Proposed	w/o Disentangled Representation	w/o Coarse-to-Fine Pipeline
SI-MSE	**0.2486**	0.2797	0.2707
ρ	**0.7600**	0.7136	0.7117

**Table 3:** Quantitative comparison of our method and other methods using the dataset of Berman et al. [35]. FT represents a fine-tuned (FT) underwater model. Ours-C is the method proposed in this section.

	DCP	UDCP	Berman et al.	UW-Net	UW-Net(FT)	Ours-C	Ours-C(FT)
SI-MSE	1.3618	0.6966	0.6755	0.4404	0.3708	0.3526	**0.2447**
ρ	0.2968	0.4894	0.6448	0.6202	0.6451	0.6823	**0.7423**

## References

[1] Eigen D., Puhrsch C., Fergus R. (2014). Depth map prediction from a single image using a multi-scale deep network. Adv. Neural Inf. Process. Syst..

[2] Chi S., Xie Z., Chen W. (2016). A laser line auto-scanning system for underwater 3D reconstruction. Sensors.

[3] Palomer A., Ridao P., Ribas D., Forest J. (2017). Underwater 3D laser scanners: The deformation of the plane. Sensing and Control for Autonomous Vehicles.

[4] Xi Q., Rauschenbach T., Daoliang L. (2017). Review of underwater machine vision technology and its applications. Mar. Technol. Soc. J..

[5] Dancu A., Fourgeaud M., Franjcic Z., Avetisyan R. (2014). Underwater reconstruction using depth sensors. SIGGRAPH ASIA Technical Briefs.

[6] Churnside J.H., Marchbanks R.D., Lembke C., Beckler J. (2017). Optical backscattering measured by airborne lidar and underwater glider. Remote Sens..

[7] Deris A., Trigonis I., Aravanis A., Stathopoulou E. (2017). Depth cameras on UAVs: A first approach. Int. Arch. Photogramm. Remote Sens. Spat. Inf. Sci..

[8] Ahamed J.R., Abas P.E., De Silva L.C. (2019). Review of underwater image restoration algorithms. IET Image Process..

[9] Massot-Campos M., Oliver-Codina G. (2015). Optical sensors and methods for underwater 3D reconstruction. Sensors.

[10] Li N., Zheng Z., Zhang S., Yu Z., Zheng H., Zheng B. (2018). The synthesis of unpaired underwater images using a multistyle generative adversarial network. IEEE Access.

[11] Gupta H., Mitra K. Unsupervised single image underwater depth estimation. Proceedings of the IEEE International Conference on Image Processing (ICIP).

[12] Zhu J.Y., Park T., Isola P., Efros A.A. Unpaired image-to-image translation using cycle-consistent adversarial networks. Proceedings of the IEEE International Conference on Computer Vision.

[13] Isola P., Zhu J.Y., Zhou T., Efros A.A. Image-to-image translation with conditional adversarial networks. Proceedings of the IEEE Conference on Computer Vision and Pattern Recognition.

[14] Wang T.C., Liu M.Y., Zhu J.Y., Tao A., Kautz J., Catanzaro B. High-resolution image synthesis and semantic manipulation with conditional GANs. Proceedings of the IEEE Conference on Computer Vision and Pattern Recognition.

[15] Choi Y., Choi M., Kim M., Ha J.W., Kim S., Choo J. StarGAN: Unified generative adversarial networks for multi-domain image-to-image translation. Proceedings of the IEEE Conference on Computer Vision and Pattern Recognition.

[16] Li J., Skinner K.A., Eustice R.M., Johnson-Roberson M. (2017). WaterGAN: Unsupervised generative network to enable real-time color correction of monocular underwater images. IEEE Robot. Autom. Lett..

[17] Cao K., Peng Y.T., Cosman P.C. Underwater image restoration using deep networks to estimate background light and scene depth. Proceedings of the IEEE Southwest Symposium on Image Analysis and Interpretation.

[18] Li C., Anwar S., Porikli F. (2020). Underwater scene prior inspired deep underwater image and video enhancement. Pattern Recognit..

[19] Skinner K.A., Zhang J., Olson E.A., Johnson-Roberson M. Uwstereonet: Unsupervised learning for depth estimation and color correction of underwater stereo imagery. Proceedings of the International Conference on Robotics and Automation.

[20] Wang N., Zhou Y., Han F., Zhu H., Zheng Y. (2019). UWGAN: Underwater GAN for real-world underwater color restoration and dehazing. arXiv.

[21] Chen X., Duan Y., Houthooft R., Schulman J., Sutskever I., Abbeel P. (2016). InfoGAN: Interpretable representation learning by information maximizing generative adversarial nets. arXiv.

[22] Spurr A., Aksan E., Hilliges O. (2017). Guiding InfoGAN with semi-supervision. Joint European Conference on Machine Learning and Knowledge Discovery in Databases.

[23] Eigen D., Fergus R. Predicting depth, surface normals and semantic labels with a common multi-scale convolutional architecture. Proceedings of the IEEE International Conference on Computer Vision.

[24] Huang G., Liu Z., Van Der Maaten L., Weinberger K.Q. Densely connected convolutional networks. Proceedings of the IEEE Conference on Computer Vision and Pattern Recognition.

[25] Goodfellow I.J., Pouget-Abadie J., Mirza M., Xu B., Warde-Farley D., Ozair S., Courville A., Bengio Y. Generative adversarial nets. Proceedings of the International Conference on Neural Information Processing Systems.

[26] Mao X., Li Q., Xie H., Lau R.Y., Wang Z., Paul Smolley S. Least squares generative adversarial networks. Proceedings of the IEEE International Conference on Computer Vision.

[27] Wang C., Xu C., Wang C., Tao D. (2018). Perceptual adversarial networks for image-to-image transformation. IEEE Trans. Image Process..

[28] Simonyan K., Zisserman A. Very deep convolutional networks for large-scale image recognition. Proceedings of the International Conference on Learning Representations.

[29] Kupyn O., Martyniuk T., Wu J., Wang Z. DeblurGAN-v2: Deblurring (orders-of-magnitude) faster and better. Proceedings of the IEEE/CVF International Conference on Computer Vision.

[30] Choi Y., Uh Y., Yoo J., Ha J.W. StarGAN v2: Diverse image synthesis for multiple domains. Proceedings of the IEEE/CVF Conference on Computer Vision and Pattern Recognition.

[31] Hu J., Ozay M., Zhang Y., Okatani T. Revisiting single image depth estimation: Toward higher resolution maps with accurate object boundaries. Proceedings of the IEEE Winter Conference on Applications of Computer Vision (WACV).

[32] Abady L., Barni M., Garzelli A., Tondi B. (2020). GAN generation of synthetic multispectral satellite images. Image and Signal Processing for Remote Sensing XXVI.

[33] He K., Sun J., Tang X. (2010). Single image haze removal using dark channel prior. IEEE Trans. Pattern Anal. Mach. Intell..

[34] Drews P.L., Nascimento E.R., Botelho S.S., Campos M.F.M. (2016). Underwater depth estimation and image restoration based on single images. IEEE Comput. Graph. Appl..

[35] Berman D., Treibitz T., Avidan S. Diving into haze-lines: Color restoration of underwater images. Proceedings of the British Machine Vision Conference (BMVC).

[36] Ancuti C., Ancuti C.O., De Vleeschouwer C. D-hazy: A dataset to evaluate quantitatively dehazing algorithms. Proceedings of the IEEE International Conference on Image Processing (ICIP).

[37] Xiao J., Hays J., Ehinger K.A., Oliva A., Torralba A. Sun database: Large-scale scene recognition from abbey to zoo. Proceedings of the IEEE Computer Society Conference on Computer Vision and Pattern Recognition.

[38] Miyato T., Kataoka T., Koyama M., Yoshida Y. Spectral normalization for generative adversarial networks. Proceedings of the International Conference on Learning Representations.

[39] Brock A., Donahue J., Simonyan K. Large scale GAN training for high fidelity natural image synthesis. Proceedings of the International Conference on Learning Representations.

[40] Zhang H., Goodfellow I., Metaxas D., Odena A. Self-attention generative adversarial networks. Proceedings of the International Conference on Machine Learning.

[41] Silberman N., Hoiem D., Kohli P., Fergus R. (2012). Indoor segmentation and support inference from rgbd images. European Conference on Computer Vision.

[42] Chen Y., Li W., Sakaridis C., Dai D., Van Gool L. Domain adaptive faster R-CNN for object detection in the wild. Proceedings of the IEEE Conference on Computer Vision and Pattern Recognition.

